# Cytomegalovirus as a Novel Target for Immunotherapy of Glioblastoma Multiforme

**DOI:** 10.3389/fonc.2014.00275

**Published:** 2014-10-07

**Authors:** Andrea Schuessler, David G. Walker, Rajiv Khanna

**Affiliations:** ^1^QIMR Berghofer Centre for Immunotherapy and Vaccine Development, QIMR Berghofer Medical Research Institute, Brisbane, QLD, Australia; ^2^BrizBrain and Spine, Newro Foundation, Wesley Hospital, Brisbane, QLD, Australia

**Keywords:** glioblastoma multiforme, immunotherapy, cytomegalovirus, T cell, adoptive transfer, checkpoint modulators

## Abstract

Progress in the treatment of glioblastoma multiforme (GBM) over the last few decades has remained marginal and GBM is still universally fatal with short survival times after initial diagnosis. Much research is focused on finding new therapeutics for GBM and immune-based approaches have shown great promise. The detection of cytomegalovirus (CMV) antigens in malignant cells has suggested that treatment strategies based on immunological intervention, such as adoptive transfer of antiviral T cells or vaccination with viral epitopes, could be exploited as cancer therapy. Here, we review the rationale for using CMV as a therapeutic target and discuss the first clinical evidence for safety and efficacy of CMV-specific cellular immunotherapy for GBM.

## Introduction

Temozolomide was the last drug to bring a significant improvement of survival for patients with glioblastoma multiforme (GBM) brain cancer (1). Now, almost a decade later, further major advances in drug development have remained elusive. GBM continues to be the most aggressive human brain cancer with 5 year survival rates below 10% (2). Standard of care treatment includes surgical resection followed by radiation and chemotherapy, however, even with optimal treatment median survival is only 15 months (1). Glioblastomas are incurable and inevitably recur with a median survival of only 6 months (3). There is an urgent need to find novel therapeutic targets and develop new treatment strategies. Immune-based approaches have great potential, in particular, because they have the great advantage of being safer and less toxic than chemotherapy drugs (4). Bevacizumab (brand name Avastin) is the only immunotherapy drug currently approved for the treatment of recurrent GBM. Bevacizumab is a monoclonal antibody that blocks vascular endothelial growth factor (VEGF), thereby reducing angiogenesis. Recently, two large clinical studies evaluating bevacizumab for the treatment of primary GBM indicated a prolonged progression free survival but failed to show a significant benefit for overall survival (5, 6). Glioblastomas are very heterogeneous tumors and it is likely that a single agent will be insufficient to achieve therapeutic benefit in a majority of patients. More than 30 studies are currently underway to assess the potential of combination therapies using bevacizumab together with radiation or other chemotherapy drugs (7). Most cellular immunotherapies for GBM under investigation at the moment focus on using tumor lysate loaded dendritic cells (DCs) or vaccination using tumor peptides [for an overview see recent review in Ref. (4)]. Preliminary results have shown some promise, however, further optimization is needed to improve anti-tumor immune responses.

## Cytomegalovirus as a Target for GBM Immunotherapy

The first report of detection of cytomegalovirus (CMV) antigens in histological sections of GBM (8) was received with much controversy. Subsequent studies have disputed (9–11) as well as confirmed (12–14) the original finding. While CMV sequences have been detected in GBM tissues (15, 16), the topic continues to be controversial as recent deep sequencing studies fail to detect CMV in GBM (17, 18). Interestingly, a clinical study evaluating vaccination using DCs pulsed with autologous tumor lysate described a patient that developed enhanced CMV-specific T cell responses after one dose of the vaccine. No such response was found in a patient with a CMV negative tumor enrolled into the same study, which provided the first immunological evidence for the presence of CMV antigens in glioma cells (19). Specific killing of primary GBM cells by autologous CMV-specific T cells was demonstrated recently and further argues for the existence of CMV antigens in brain tumors (20). The precise role of CMV in GBM remains unclear. In contrast to other members of the herpes virus family like Epstein–Barr virus, CMV is not an oncogenic virus. However, CMV encodes many genes that may enable hallmarks of cancer such as pro-angiogenic signaling, immune evasion, and deregulation of the cell cycle (21–26). From a therapeutic perspective, the presence of CMV antigens provides a unique opportunity to exploit pre-existing antiviral immunity for immune-based GBM treatment. Further support for this rationale stems from the finding that lower levels of CMV antigens in GBM sections are associated with prolonged survival (27, 28). Therapeutic use of antiviral drug valganciclovir for GBM patients has been explored without major side effects, but further studies are needed to assess efficacy (29–31). In the context of immunotherapy, a previous study of CMV-specific T cell responses in GBM patients has detected functional impairment of antiviral T cells. Despite similar frequencies in GBM patients and healthy individuals, CMV-specific cytotoxic T cells in GBM patients showed limited ability to produce multiple cytokines (macrophage inflammatory protein MIP-1β, tumor necrosis factor TNF, interferon IFNγ) and to mobilize CD107a in response to CMV epitopes (32). Importantly, polyfunctionality of CMV-specific T cells isolated from GBM patients could be restored by *in vitro* stimulation with CMV antigens and γC cytokines (32). This suggests that adoptive transfer of *in vitro* expanded T cells could improve CMV-specific immune responses in GBM patients. Indeed, this preliminary study has shown that immunotherapy using CMV-specific T cells was coincident with prolonged survival in one patient (32). CMV-specific immunity is characterized by high frequencies of CMV-specific cytotoxic T cells in seropositive individuals. While tumor associated antigens are usually poorly immunogenic, viral antigens provide a strong stimulus that makes expansion of high-frequency antiviral T cell cultures comparatively easy. Consequently, several laboratories have established efficient protocols for the *in vitro* expansion of CMV-specific cytotoxic T cells from GBM patients for the purpose of immunotherapy (20, 32, 33). Efficient killing of autologous primary tumor cells by CMV-specific T cells was demonstrated *in vitro*, thus, providing direct evidence that CMV-specific cytotoxic T cells can be applied for GBM therapy (20).

## Current Investigations of Cytomegalovirus Specific GBM Immunotherapy

Two clinical studies using CMV-specific immunotherapy are currently recruiting participants and two further studies using CMV-specific autologous lymphocyte transfer and DC vaccination have completed enrollment (Table 1). Of the currently recruiting studies, one study is testing genetically modified CMV-specific cytotoxic cells for the treatment of recurrent GBM (clinical trials identifier NCT01109095). CMV-specific T cells are engineered to express a chimeric antigen receptor (CAR) recognizing human epidermal growth factor receptor 2 (HER-2) coupled to CD28 (7). HER-2 antigen is expressed on a majority of GBM cells and CD28 promotes sustained T cell activity. While the primary objective of the study is safety, it might provide some insight if the targeting of glioma cells using T cells specific for CMV and HER-2 can provide a survival benefit. The second CMV-specific GBM immunotherapy under investigation is based on a combination of DC vaccination and a monoclonal antibody directed against CD25, which blocks interleukin IL-2 signaling (clinical trials identifier NCT00626483). Autologous DCs are loaded with CMV pp65-LAMP mRNA and administered with different doses of CD25 antibody, which is expected to inhibit regulatory T cells (7, 34). In this approach, alleviating the suppressive effect of regulatory T cells might lead to more efficient CMV-specific T cell activity. Results from these studies are expected by 2016 and 2015, respectively.

**Table 1:** **Current clinical trials evaluating CMV-specific immunotherapy for GBM**.

Intervention	GBM type	Enrollment	Phase	Duration	NCT number	Status
Genetically modified HER.CAR CMV-specific CTLs	Recurrent	18	I	2010–2031	NCT01109095	Recruiting
DC vaccine (CMV pp65-LAMP mRNA loaded DC), basiliximab (anti-CD25)	Primary	18	I	2007–2015	NCT00626483	Recruiting
DC vaccine (CMV pp65-LAMP mRNA loaded DC) with or without autologous lymphocyte transfer, tetanus toxoid	Primary	16	I	2006–2016	NCT00639639	Active, not recruiting
CMV autologous lymphocyte transfer with or without DC vaccine (CMV pp65-LAMP mRNA loaded DC)	Primary	12	I	2008–2016	NCT00693095	Active, not recruiting

## First Clinical Outcomes of Cytomegalovirus Specific Therapy for GBM

The first formal clinical assessment of CMV-specific adoptive T cell immunotherapy was completed recently (35). Ten CMV seropositive patients with recurrent GBM received three or four infusions of autologous CMV-specific cytotoxic T cells generated following *in vitro* stimulation with synthetic CMV epitopes. The treatment was shown to be safe and only mild side effects were recorded. While the patient cohort was too small to draw definite conclusions about efficacy and effects on overall survival, it is notable that 4 out of 10 patients remained completely disease free during the study period. At present, the follow up time for these patients since initiation of T cell therapy ranges from 10 months to more than 4 years, which extends well beyond the expected survival median time of 6 months after tumor recurrence. Immunological and molecular analysis revealed a number of important insights. Analysis of a range of immunological parameters on peripheral blood mononuclear cells (PBMC) before and after T cell therapy failed to reveal major changes. In contrast, molecular analysis of the T cell product used for adoptive therapy showed a signature of seven genes (EOMES, IFNG, BCL6, XAF1, CCL5, CTLA-4, FOXP3) that distinguished individuals with long-term progression free survival from patients that progressed more rapidly. This signature was consistent with T cell activation (i.e., upregulation of T cell transcription factor Eomes and effector molecule IFNγ, downregulation of inhibitory receptor CTLA-4) suggesting that more functional CMV-specific T cells are more efficient in controlling cancer relapse. One patient had a tumor recurrence after therapy and isolation of T cells from the resected tissue revealed the first evidence that CMV-specific T cells are present in tumor tissues. In addition, intratumoral T cells were found to express higher levels of immune inhibitory molecules, indicating that local immunosuppression might be an important factor in the development of effective therapies.

## Immune Regulatory Mechanisms to Consider

Immunosuppression is one of the classic hallmarks of human cancers (36). In the context of GBM, this principle applies locally in the tumor microenvironment as well as systemically (4). Immune inhibitory receptors expressed on T cells such as PD-1, CTLA-4, TIM-3, or BTLA play an important role for modulation of T cell responses. Expression of PD-L1, the ligand for PD-1, has been detected in glioma samples (37) and a recent study found high-PD-L1 levels in tumor tissue to be associated with poor survival (38). Blockade of such checkpoint inhibitors might therefore be an attractive treatment option to boost intrinsic tumor defenses. Interference with the PD-1/PD-L1 interaction by monoclonal antibodies directed against PD-1 is currently under clinical investigation for the treatment of recurrent GBM (clinical trials identifier NCT01952769). Similarly, a monoclonal antibody blocking CTLA-4 (ipilimumab) that is currently approved for the treatment of metastatic melanoma might be effective in enhancing anti glioma immune responses. A combination therapy of anti-VEGF and anti-PD-1 or anti-CTLA-4 is in phase II testing for recurrent GBM (clinical trials identifier NCT02017717). In the context of CMV-specific immunotherapy for GBM, analysis of intratumoral CMV-specific T cells in a patient that relapsed after T cell therapy indicated that local immunosuppression might have contributed to treatment failure (35). While further testing is needed to confirm this finding in a larger patient cohort, it is tempting to speculate that combination of CMV-specific T cells with blockade of inhibitory receptors might boost efficacy of adoptive immunotherapy.

## Conclusion and Perspective

**Figure 1:**
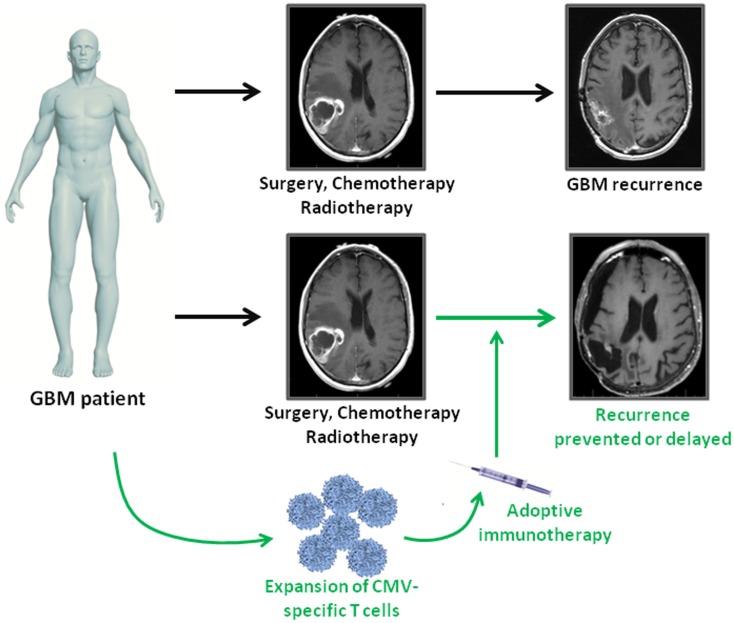
**Adoptive immunotherapy as an addition to standard treatment for GBM patients**. GBM patients receive standard of care therapy consisting of surgery followed by chemo- and radiotherapy. However, tumor recurrence is inevitable. In GBM patients, who are CMV seropositive, virus-specific T cell immunotherapy can be used as consolidative treatment to enhance intrinsic immune responses against viral antigen expressing tumor cells. If effective, adoptive immunotherapy has the potential to prolong progression free survival or even prevent relapse in GBM patients.

Immunotherapies provide a novel approach to complement standard therapies for GBM treatment (Figure 1). CMV antigens provide an attractive target for cellular immunotherapies that could provide more efficient tumor recognition than tumor associated antigens. The first clinical assessment of CMV-specific T cell therapy has been completed and proved to be safe with potential clinical benefit. Further clinical trials using CMV directed immunotherapy for GBM are underway. Cancer associated immunosuppression has to be taken into account as it might limit the effectiveness of antiviral T cells within the tumor tissue. Future studies should therefore focus on multimodal strategies combining cellular immunotherapy with blockage of inhibitory receptors on T cells.

## Conflict of Interest Statement

The authors declare that the research was conducted in the absence of any commercial or financial relationships that could be construed as a potential conflict of interest.

## References

[1] Stupp R Mason WP van den Bent MJ Weller M Fisher B Taphoorn MJ Radiotherapy plus concomitant and adjuvant temozolomide for glioblastoma. N Engl J Med (2005) 352(10):987–96 10.1056/NEJMoa043330 15758009

[2] Stupp R Hegi ME Mason WP van den Bent MJ Taphoorn MJ Janzer RC Effects of radiotherapy with concomitant and adjuvant temozolomide versus radiotherapy alone on survival in glioblastoma in a randomised phase III study: 5-year analysis of the EORTC-NCIC trial. Lancet Oncol (2009) 10(5):459–66 10.1016/S1470-2045(09)70025-7 19269895

[3] Reardon DA Herndon JE II Peters KB Desjardins A Coan A Lou E Bevacizumab continuation beyond initial bevacizumab progression among recurrent glioblastoma patients. Br J Cancer (2012) 107(9):1481–7 10.1038/bjc.2012.415 23037712 PMC3493761

[4] Reardon DA Wucherpfennig KW Freeman G Wu CJ Chiocca EA Wen PY An update on vaccine therapy and other immunotherapeutic approaches for glioblastoma. Expert Rev Vaccines (2013) 12(6):597–615 10.1586/erv.13.41 23750791 PMC3982399

[5] Chinot OL Wick W Mason W Henriksson R Saran F Nishikawa R Bevacizumab plus radiotherapy-temozolomide for newly diagnosed glioblastoma. N Engl J Med (2014) 370(8):709–22 10.1056/NEJMoa1308345 24552318

[6] Gilbert MR Dignam JJ Armstrong TS Wefel JS Blumenthal DT Vogelbaum MA A randomized trial of bevacizumab for newly diagnosed glioblastoma. N Engl J Med (2014) 370(8):699–708 10.1056/NEJMoa1308573 24552317 PMC4201043

[7] ClinicalTrials.gov. Available from: www.clinicaltrials.gov

[8] Cobbs CS Harkins L Samanta M Gillespie GY Bharara S King PH Human cytomegalovirus infection and expression in human malignant glioma. Cancer Res (2002) 62(12):3347–50 12067971

[9] Poltermann S Schlehofer B Steindorf K Schnitzler P Geletneky K Schlehofer JR Lack of association of herpesviruses with brain tumors. J Neurovirol (2006) 12(2):90–9 10.1080/13550280600654573 16798670

[10] Sabatier J Uro-Coste E Pommepuy I Labrousse F Allart S Tremoulet M Detection of human cytomegalovirus genome and gene products in central nervous system tumours. Br J Cancer (2005) 92(4):747–50 10.1038/sj.bjc.6602339 15700045 PMC2361882

[11] Lau SK Chen YY Chen WG Diamond DJ Mamelak AN Zaia JA Lack of association of cytomegalovirus with human brain tumors. Mod Pathol (2005) 18(6):838–43 10.1038/modpathol.3800352 15578071

[12] Lucas KG Bao L Bruggeman R Dunham K Specht C The detection of CMV pp65 and IE1 in glioblastoma multiforme. J Neurooncol (2011) 103(2):231–8 10.1007/s11060-010-0383-6 20820869

[13] Mitchell DA Xie W Schmittling R Learn C Friedman A McLendon RE Sensitive detection of human cytomegalovirus in tumors and peripheral blood of patients diagnosed with glioblastoma. Neuro Oncol (2008) 10(1):10–8 10.1215/15228517-2007-035 17951512 PMC2600830

[14] Scheurer ME Bondy ML Aldape KD Albrecht T El-Zein R Detection of human cytomegalovirus in different histological types of gliomas. Acta Neuropathol (2008) 116(1):79–86 10.1007/s00401-008-0359-1 18351367 PMC3001277

[15] Bhattacharjee B Renzette N Kowalik TF Genetic analysis of cytomegalovirus in malignant gliomas. J Virol (2012) 86(12):6815–24 10.1128/JVI.00015-12 22496213 PMC3393585

[16] Ranganathan P Clark PA Kuo JS Salamat MS Kalejta RF Significant association of multiple human cytomegalovirus genomic loci with glioblastoma multiforme samples. J Virol (2012) 86(2):854–64 10.1128/JVI.06097-11 22090104 PMC3255835

[17] Cimino PJ Zhao G Wang D Sehn JK Lewis JS Jr Duncavage EJ Detection of viral pathogens in high grade gliomas from unmapped next-generation sequencing data. Exp Mol Pathol (2014) 96(3):310–5 10.1016/j.yexmp.2014.03.010 24704430

[18] Cosset E Petty TJ Dutoit V Cordey S Padioleau I Otten-Hernandez P Comprehensive metagenomic analysis of glioblastoma reveals absence of known virus despite antiviral-like type I interferon gene response. Int J Cancer (2013) 135(6):1381–9 10.1002/ijc.28670 24347514 PMC4235296

[19] Prins RM Cloughesy TF Liau LM Cytomegalovirus immunity after vaccination with autologous glioblastoma lysate. N Engl J Med (2008) 359(5):539–41 10.1056/NEJMc0804818 18669440 PMC2775501

[20] Nair SK De Leon G Boczkowski D Schmittling R Xie W Staats J Recognition and killing of autologous, primary glioblastoma tumor cells by human cytomegalovirus pp65-specific cytotoxic T cells. Clin Cancer Res (2014) 20(10):2684–94 10.1158/1078-0432.CCR-13-3268 24658154 PMC4059212

[21] Noriega V Redmann V Gardner T Tortorella D Diverse immune evasion strategies by human cytomegalovirus. Immunol Res (2012) 54(1–3):140–51 10.1007/s12026-012-8304-8 22454101

[22] Smith C Khanna R Immune regulation of human herpesviruses and its implications for human transplantation. Am J Transplant (2013) 13(Suppl 3):9–23 10.1111/ajt.12005 23347211

[23] Maussang D Langemeijer E Fitzsimons CP Stigter-van Walsum M Dijkman R Borg MK The human cytomegalovirus-encoded chemokine receptor US28 promotes angiogenesis and tumor formation via cyclooxygenase-2. Cancer Res (2009) 69(7):2861–9 10.1158/0008-5472.CAN-08-2487 19318580

[24] Slinger E Maussang D Schreiber A Siderius M Rahbar A Fraile-Ramos A HCMV-encoded chemokine receptor US28 mediates proliferative signaling through the IL-6-STAT3 axis. Sci Signal (2010) 3(133):ra58 10.1126/scisignal.2001180 20682912

[25] Soroceanu L Cobbs CS Is HCMV a tumor promoter? Virus Res (2011) 157(2):193–203 10.1016/j.virusres.2010.10.026 21036194 PMC3082728

[26] Soroceanu L Matlaf L Bezrookove V Harkins L Martinez R Greene M Human cytomegalovirus US28 found in glioblastoma promotes an invasive and angiogenic phenotype. Cancer Res (2011) 71(21):6643–53 10.1158/0008-5472.CAN-11-0744 21900396 PMC3206211

[27] Rahbar A Orrego A Peredo I Dzabic M Wolmer-Solberg N Straat K Human cytomegalovirus infection levels in glioblastoma multiforme are of prognostic value for survival. J Clin Virol (2013) 57(1):36–42 10.1016/j.jcv.2012.12.018 23391370

[28] Rahbar A Stragliotto G Orrego A Peredo I Taher C Willems J Low levels of human cytomegalovirus infection in glioblastoma multiforme associates with patient survival – a case-control study. Herpesviridae (2012) 3:3 10.1186/2042-4280-3-3 22424569 PMC3348037

[29] Soderberg-Naucler C Rahbar A Stragliotto G Survival in patients with glioblastoma receiving valganciclovir. N Engl J Med (2013) 369(10):985–6 10.1056/NEJMc1302145 24004141

[30] Stragliotto G Rahbar A Solberg NW Lilja A Taher C Orrego A Effects of valganciclovir as an add-on therapy in patients with cytomegalovirus-positive glioblastoma: a randomized, double-blind, hypothesis-generating study. Int J Cancer (2013) 133(5):1204–13 10.1002/ijc.28111 23404447

[31] Cobbs CS Does valganciclovir have a role in glioblastoma therapy? Neuro Oncol (2014) 16(3):330–1 10.1093/neuonc/nou009 24523453 PMC3922528

[32] Crough T Beagley L Smith C Jones L Walker DG Khanna R Ex vivo functional analysis, expansion and adoptive transfer of cytomegalovirus-specific T-cells in patients with glioblastoma multiforme. Immunol Cell Biol (2012) 90(9):872–80 10.1038/icb.2012.19 22508289

[33] Ghazi A Ashoori A Hanley PJ Brawley VS Shaffer DR Kew Y Generation of polyclonal CMV-specific T cells for the adoptive immunotherapy of glioblastoma. J Immunother (2012) 35(2):159–68 10.1097/CJI.0b013e318247642f 22306904 PMC3280423

[34] Mitchell DA Cui X Schmittling RJ Sanchez-Perez L Snyder DJ Congdon KL Monoclonal antibody blockade of IL-2 receptor alpha during lymphopenia selectively depletes regulatory T cells in mice and humans. Blood (2011) 118(11):3003–12 10.1182/blood-2011-02-334565 21768296 PMC3175779

[35] Schuessler A Smith C Beagley L Boyle GM Rehan S Matthews K Autologous T cell therapy for cytomegalovirus as a consolidative treatment for recurrent glioblastoma. Cancer Res (2014) 74(13):3466–76 10.1158/0008-5472.CAN-14-0296 24795429

[36] Hanahan D Weinberg RA Hallmarks of cancer: the next generation. Cell (2011) 144(5):646–74 10.1016/j.cell.2011.02.013 21376230

[37] Jacobs JF Idema AJ Bol KF Nierkens S Grauer OM Wesseling P Regulatory T cells and the PD-L1/PD-1 pathway mediate immune suppression in malignant human brain tumors. Neuro Oncol (2009) 11(4):394–402 10.1215/15228517-2008-104 19028999 PMC2743219

[38] Liu Y Carlsson R Ambjorn M Hasan M Badn W Darabi A PD-L1 expression by neurons nearby tumors indicates better prognosis in glioblastoma patients. J Neurosci (2013) 33(35):14231–45 10.1523/JNEUROSCI.5812-12.2013 23986257 PMC6618508

